# Prediction of Bandgap in Lithium-Ion Battery Materials Based on Explainable Boosting Machine Learning Techniques

**DOI:** 10.3390/ma17246217

**Published:** 2024-12-19

**Authors:** Haobo Qin, Yanchao Zhang, Zhaofeng Guo, Shuhuan Wang, Dingguo Zhao, Yuekai Xue

**Affiliations:** 1 Department of Resources and Environmental Engineering, Hebei Vocational University of Technology and Engineering, Xingtai 054000, China; qhbzzz777@163.com (H.Q.); guozhaofeng@xpc.edu.cn (Z.G.); 2 College of Metallurgy and Energy, North China University of Science and Technology, Tangshan 063000, China; wshh88@ncst.edu.cn (S.W.); gyyzhao@163.com (D.Z.)

**Keywords:** silicon oxides, bandgap, material discovery, machine learning

## Abstract

The bandgap is a critical factor influencing the energy density of batteries and a key physical quantity that determines the semiconducting behavior of materials. To further improve the prediction accuracy of the bandgap in silicon oxide lithium-ion battery materials, a boosting machine learning model was established to predict the material’s bandgap. The optimal model, AdaBoost, was selected, and the SHapley Additive exPlanations (SHAP) method was used to quantitatively analyze the importance of different input features in relation to the model’s prediction accuracy. It was found that AdaBoost performed exceptionally well in terms of prediction accuracy, ranking as the best among five predictive models. Using the SHAP method to interpret the AdaBoost model, it was discovered that there is a significant positive correlation between the energy of the conduction band minimum (cbm) of silicon oxides and the bandgap, with the bandgap size showing an increasing trend as the cbm rises. Additionally, the study revealed a strong negative correlation between the Fermi level of silicon oxides and the bandgap, with the bandgap expanding as the Fermi level decreases. This research demonstrates that boosting-type machine learning models perform superiorly in predicting the bandgap of silicon oxide materials.

## 1. Introduction

Lithium-ion batteries, characterized by their high energy density, absence of memory effect, long service life, reasonable price, and environmental friendliness, have become the most widely used secondary batteries, extensively applied in consumer electronics and portable electronic devices [1,2]. However, existing battery systems fail to meet the growing demand for high energy density in lithium-ion applications [3,4,5]. Enhancing the energy density of lithium-ion batteries while ensuring safety and cycling performance has become a focal point of research [6]. Developing high specific capacity battery materials is crucial to addressing the aforementioned issues [7]. Silicon oxides have attracted considerable attention in the energy storage field due to their abundant reserves, low cost, ease of synthesis, suitable power density, and high energy density [8,9,10]. However, silicon oxides still have room for improvement due to their lower initial Coulombic efficiency and poorer electronic conductivity [11,12].

The bandgap is a critical factor affecting the energy density of batteries that is directly related to the electronic structure and chemical activity of battery materials [13,14]. The electrochemical performance of silicon oxide lithium-ion battery materials is closely related to their oxygen content. As the oxygen content increases, the cycle stability of the electrode materials improves, but the initial Coulombic efficiency and battery capacity decrease [15]. Therefore, precise control of the bandgap in silicon oxides not only effectively adjusts their electronic structure and chemical activity but also enhances the initial Coulombic efficiency and battery capacity while maintaining the battery’s cycle stability, which is essential for improving the energy density of batteries [16]. Meanwhile, as a key physical property determining the semiconducting behavior of materials, the bandgap plays a decisive role in evaluating the application prospects of materials in multiple fields, such as photovoltaic conversion, catalytic activity, and sensor response [17,18]. Consequently, elucidating the intrinsic relationship between the bandgap of silicon oxides and their chemical composition and microstructure has also become an urgent issue in current materials science research.

With the rapid development of artificial intelligence technology and the construction and popularization of material properties databases, machine learning has been applied to various fields including medicine and materials science [19,20,21]. Petti et al. [22] evaluated the performance of various machine learning methods in predicting organ utilization, demonstrating that XGBoost achieved the best performance in predicting liver and kidney organ usage, with AUC-ROC scores of 0.925 and 0.952, respectively. The XGBoost method showed significant improvements in predicting the discarding of donor allografts for both kidneys and livers in solid organ transplantation procedures. Ghafouri-Kesbi et al. [23] utilized three machine learning algorithms and genomic best linear unbiased prediction (GBLUP) to predict genomic breeding values (GBVs). Their experiments showed that GBLUP outperformed machine learning methods in prediction accuracy when the number of quantitative trait loci (QTLs) was large and their effects were normally distributed. In terms of mean square error (MSE) for GBV predictions, boosting machine learning methods generally outperformed other approaches. Jafari et al. [24] proposed a method based on ensemble boosting algorithms to predict the state of charge (SOC) of batteries, using machine learning models to analyze the nonlinear mapping between voltage and current values. The study indicated that the extreme gradient boosting algorithm could generate faster and more accurate estimates for SOC applications, with both simulations and experiments confirming its accuracy in estimating SOC. Commonly used machine learning models include support vector machines, decision trees, random forests, and artificial neural networks, among others [25].

In recent years, predicting material properties using machine learning models to guide targeted material development has become a new research hotspot in materials science [26,27,28]. Wang et al. [29] predicted the unconfined compressive strength of cement fly ash mortar using boosting machine learning methods and compared the prediction accuracy of the optimal boosting machine learning model with other commonly used machine learning models. Zhuo et al. [30] accurately predicted the bandgap of inorganic solids based on their composition using a support vector regression model, with predictions closer to experimentally reported values than those calculated using density functional theory (DFT). Wang et al. [31] employed a stacking method to integrate ten baseline models for predicting material bandgaps. The study showed that the stacking model achieved the highest R^2^ value in benchmark datasets, demonstrating the excellent performance of the stacking method in bandgap regression. Venkatraman [32] employed machine learning models to predict the bandgaps of various materials and compared these predictions with those obtained from a series of DFT functionals specifically designed for bandgap determination. The study found that machine learning models typically yield lower prediction errors. Moreover, ML methods offer advantages in speed and ease of use over DFT approaches, making them suitable for informatics-assisted bandgap engineering. Wu et al. [33] adopted a data-driven approach to establish a database containing the bandgaps of 53,361 mixed ABX3 compounds and evaluated halide segregation. The study found that the bandgap increases as the ionic radii of the A-site and X-site decrease. This composition-dependent variation in bandgap promotes halide segregation. Meredig et al. [34] introduced an innovative machine learning approach for combinatorial screening of new materials within an unconstrained compositional space. By developing a machine learning model trained on thousands of DFT calculation results, this study was able to predict the thermodynamic stability of materials in a shorter timeframe and successfully predicted approximately 4500 novel stable ternary compounds. The high-ranking predictions were validated using DFT crystal structure prediction, confirming eight new compounds including SiYb_3_F_5_, Pa_2_O(SiO_6_), and S_2_(VF_6_). This method significantly accelerates the pace of material exploration and strongly supports data-driven material development. It can be widely applied to material research tasks such as predicting lithium capacity, bandgaps, or magnetic moments. This provides inspiration for our work.

The studies by the aforementioned scholars have demonstrated the feasibility of using machine learning methods to predict material bandgaps, showing promising results. However, existing models still have room for improvement in terms of prediction accuracy and generalization ability. Studies have shown that ensemble machine learning models have advantages over traditional machine learning models in terms of prediction accuracy [35]. As a common type of ensemble machine learning model, boosting models have been widely applied. However, there is a lack of research focusing on the prediction of silicon oxide bandgaps and the promotion of directed optimization of silicon oxide lithium-ion battery materials’ performance using machine learning, and the application of boosting machine learning models in this area is also limited. Moreover, machine learning models are often considered “black box” models; despite their good performance in prediction tasks, they cannot be easily explained, which limits their application in the directed development of materials. Lundberg and Lee [36] developed the SHapley Additive exPlanations (SHAP) framework for explaining machine learning models, which allows for the quantitative evaluation of each feature’s contribution to the prediction of target values.

To further enhance the prediction accuracy of the bandgap characteristics of silicon oxide lithium-ion battery materials and to enable rapid screening of novel functional materials, three boosting machine learning algorithms and two traditional machine learning models were employed to predict the bandgap of silicon oxide materials, including adaptive boosting (AdaBoost) [37], gradient boosting regressor (GBR) [38], extreme gradient boosting (XGB) [39], random forest [40], and support vector regression (SVR) [41]. This study focuses on investigating the application performance of interpretable boosting machine learning models (AdaBoost, GBR, and XGB) in predicting the bandgap of silicon oxide materials. By comparing and analyzing with two commonly used machine learning models, the aim is to select the optimal model for predicting the bandgap of silicon oxide materials, with the expectation of optimizing the electrochemical performance of the materials through accurate prediction models. Furthermore, the SHAP method was used to interpret the best model, exploring the importance of each input feature for bandgap prediction, providing a basis for the optimization of the semiconducting properties of silicon oxide materials and offering new perspectives and strategies for material modification and performance enhancement.

## 2. Data Analysis and Feature Selection

**Figure 1:**
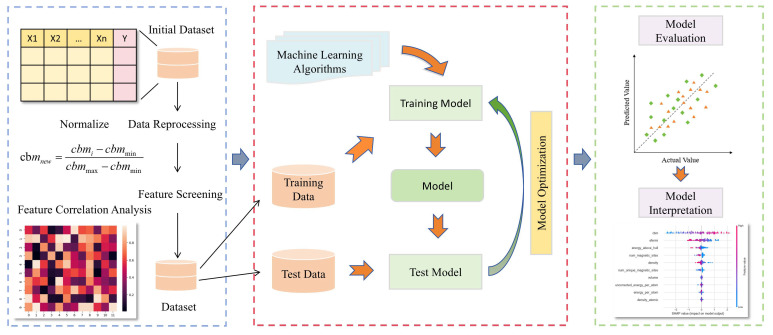
A machine learning-based framework for bandgap prediction in silicon oxide.

This paper predicts the bandgap width of silicon oxide materials by training machine learning models. Figure 1 illustrates the entire workflow of developing the model in this study, which consists of three parts. The first part involves data analysis and feature processing. An initial dataset of silicon oxide materials is established, followed by preprocessing and feature selection of the data. The data are then divided into a training dataset (80%) and a testing dataset (20%). The second part focuses on model training and optimization. The third part involves model evaluation and interpretation, where the SHAP method is used to explain the optimal model.

### 2.1. Data Analysis

To investigate the intrinsic relationship between the bandgap width of silicon oxides and their material performance and to accelerate the development of silicon-containing oxide materials using data-driven approaches, we retrieved silicon oxide data from Materials Project [42,43], an open-source database based on high-throughput computations. The dataset consists of 7637 entries with ten features each. The input features of the dataset include formula_pretty, volume, cbm, density, density_atomic, efermi, num_magnetic_sites, and num_unique_magnetic_sites, while band_gap serves as the output parameter of the model. All data utilized in this study are entirely based on computational results from Materials Project. These results were obtained using standardized high-throughput computational methods rather than experimental measurements. Consequently, due to the limitations of DFT calculations, the computed results may not be entirely accurate and only represent theoretical predictions. Data from Materials Project are typically calculated using DFT, including computational frameworks such as the generalized gradient approximation (GGA) or generalized gradient approximation + Hubbard U (GGA + U). The extracted results provide high numerical accuracy, yet such precision is unattainable in experiments. To improve readability of the data and reflect the actual precision of computations and experiments, the decimal places of each feature’s data were reasonably adjusted, as shown in part in Table 1.

To ensure data consistency and model accuracy, preprocessing was performed on the original data. Outliers in the data were detected and removed, and interpolation was used to fill in a small amount of missing data. Given that the bandgap width is highly sensitive to changes in cbm (the energy of the conduction band minimum), filling in missing values can significantly affect the model, so data records with missing values for this feature were deleted. After data cleaning, 6919 valid records were retained.

**Figure 2:**
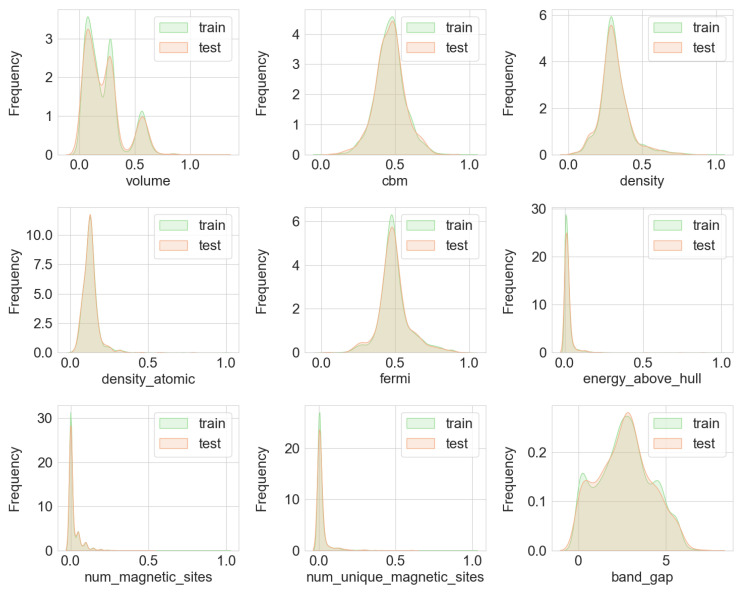
Data distribution of the training and test sets.

For the training of machine learning models, the original dataset was split into a training set (train_data) and a testing set (test_data) at a ratio of 0.8:0.2. The data distribution of the training and testing sets is shown in Figure 2. The distributions of eleven numerical features in both the training and testing sets are roughly similar and mostly conform to a normal distribution. This ensures that the trained model will have good generalization capabilities, and its performance on the testing set will accurately reflect its expected performance on new data. In this study, the bandgap was chosen as the target feature, and a model was built using machine learning methods based on the organized dataset to predict the bandgap values of silicon oxide-containing materials.

### 2.2. Feature Engineering

Feature engineering is a critical step in the machine learning process, directly impacting the performance and predictive capability of the model [44]. Combining domain knowledge and data analysis skills to effectively and reasonably process data features can significantly enhance model performance, improve model interpretability, and boost model generalization capabilities [45]. To ensure that the trained machine learning model performs well, nine features were selected based on the physicochemical properties of the materials and the structural characteristics of the compounds to describe silicon oxide materials. All features except the bandgap are input features. Specific features and their descriptions are shown in Table 2.

Due to the broad distribution of the volume feature data, spanning several orders of magnitude, and significant scale differences with other feature data, direct use in model training would affect the training and prediction performance of the model. To eliminate the influence of scale and ensure comparability between different features, the selected numerical features underwent min–max normalization [46]. Data normalization does not alter the correlations between features or the shape of the data distribution.

(1) $$cbm_{new}=\frac{cbm_{i}-cbm_{\min}}{cbm_{\max}-cbm_{\min}}$$

**Figure 3:**
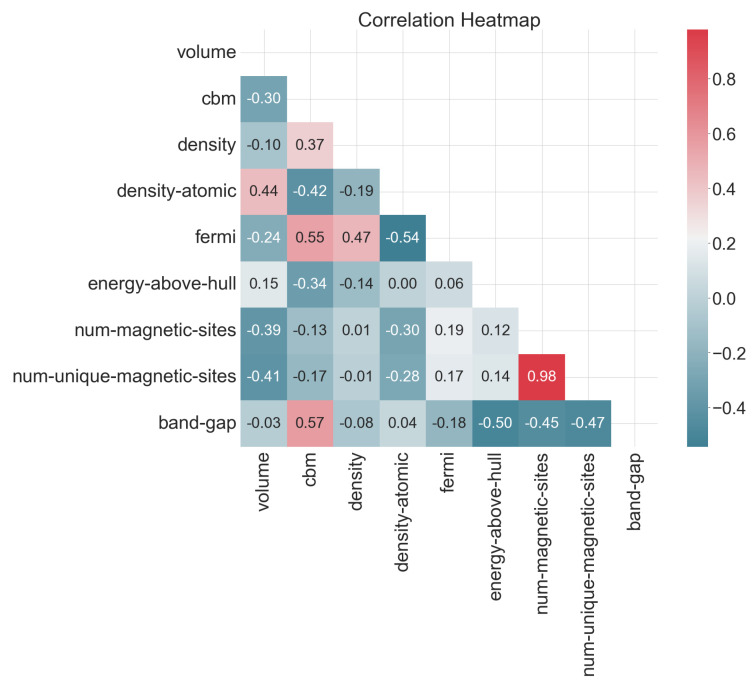
Heatmap of Pearson’s correlation coefficient for 11 features.

To reveal the factors that most significantly influence the target feature band_gap, the Pearson correlation coefficients (R values) between the selected 8 input features and the target feature were analyzed, and a Pearson correlation coefficient heatmap was created to display the relationships between the features. Red represents a positive correlation, and blue represents a negative correlation. Deeper colors indicate stronger correlations. From Figure 3, it can be seen that the R value between cbm and the target feature band_gap is 0.57, indicating a strong positive correlation. The band_gap is defined as the energy difference between the top of the valence band and the bottom of the conduction band. The size of the band_gap determines the minimum energy required for an electron to transition from the valence band to the conduction band, so cbm has a direct impact on the band_gap. The R values between energy_above_hull, num_magnetic_sites, and num_unique_magnetic_sites and band_gap are −0.5, −0.45, and −0.47, respectively, indicating strong negative correlations and consistent effects on band_gap. However, these factors do not have a direct mathematical relationship with the bandgap but indirectly affect the size and properties of the bandgap by influencing the electronic structure and interactions within the material. The remaining features have smaller R values, showing weaker positive or negative correlations, and serve to refine the model during training. Volume, density and density_atomic have weak correlations with the target feature band_gap. Using machine learning models, preliminary training was conducted on datasets both including and excluding these three features, and it was found that models trained on datasets including these three features performed better. Therefore, despite their weak correlations with the target feature, these three features are also used in subsequent model training.

### 2.3. Model Evaluation Metrics

This study evaluates the model’s performance using the coefficient of determination (R^2^), mean absolute error (MAE), and mean squared error (MSE) as evaluation criteria.

The R^2^ score is a crucial metric for evaluating model fit, quantifying the correlation between predicted and actual values to measure the model’s explanatory power over the data. An R^2^ score closer to 1 indicates a better fit of the model to the data, suggesting stronger explanatory power.

MAE and MSE are commonly used metrics to measure the magnitude of prediction errors in models. They assess the precision of model predictions by calculating the mean squared difference between predicted and actual values. Smaller values of MAE and MSE indicate better predictive performance of the model.

## 3. Results

### 3.1. Model Performance Comparison

Based on two conventional machine learning models, random forest and SVR, and three boosting machine learning models, AdaBoost, GBR, and XGB, we trained predictive models for forecasting the bandgap of silicon oxide materials using the training set data obtained from the aforementioned data processing and feature engineering. The models were validated using the test set, and their performance was evaluated using the coefficient of determination (R^2^), mean absolute error (MAE), and mean squared error (MSE) as evaluation criteria. Table 3 summarizes the evaluation metrics of the five trained models on the test set. Comparing the evaluation metrics of the five models reveals that the AdaBoost model, as a classic boosting model, exhibits superior performance in predicting the bandgap of silicon oxide materials. The AdaBoost model exhibits superior predictive capability, with an R^2^ as high as 0.93 on the test set, indicating excellent generalization ability for unseen data. Additionally, its MAE and MSE are as low as 0.21 and 0.17, respectively, performing the best among the five models, which underscores its remarkable predictive accuracy. In comparison, the random forest model’s performance is very close to that of AdaBoost. Although its R^2^ is 0.01 lower than AdaBoost, and its MAE is 0.01 lower while MSE is 0.02 higher, it still demonstrates a strong overall predictive capacity. Both the GBR and XGB models exhibit nearly identical predictive performance. Both models achieved an R^2^ of 0.91, with only a 0.01 difference in MAE and MSE, showing highly similar capabilities in fitting the data. However, compared to AdaBoost, GBR and XGB have slightly inferior overall performance. In contrast, the SVR model performs worse than the other four models, with an R^2^ of 0.89, significantly lower than the others. Moreover, its MAE and MSE are much higher than those of the other four models, indicating the poorest predictive performance in this task.

### 3.2. Model Analysis

**Figure 4:**
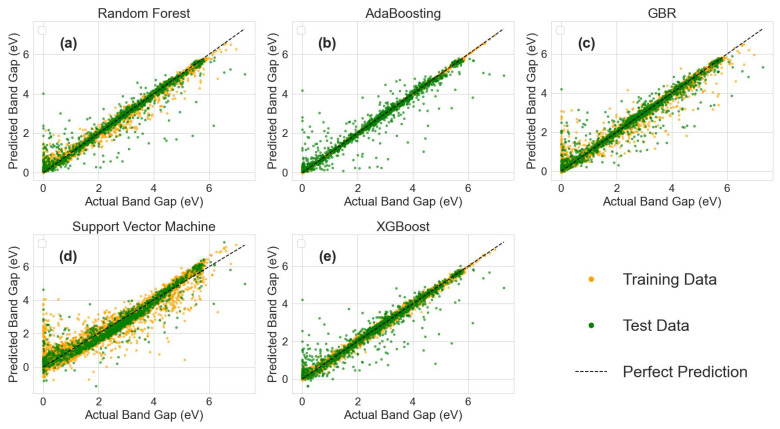
(**a**–**e**) Bandgap prediction performance of five models.

Figure 4 illustrates the training performance of the five models in predicting the bandgap on both the training and test sets. Figure 4b shows the relationship between actual and predicted values for the AdaBoost model when R^2^ is 0.93. Both the training and test set predictions cluster closely around the best-fit regression line, indicating that the AdaBoost model has strong predictive power for the bandgap and performs well in predicting unknown data, demonstrating robust generalization ability. From Figure 4e, it can be seen that the XGB model also has good predictive ability for the training set data, but its prediction performance on the test set is slightly worse than that of the AdaBoost model, indicating less robust generalization ability. The XGB model exhibits excellent predictive performance on the training set data but has larger prediction errors on the test data, suggesting that the algorithm has overlearned noise and outliers in the data, leading to an overfitting tendency. In Figure 4d, the SVR model has larger prediction errors on the training set but performs well on the test set, indicating underfitting. This may be due to insufficient training set data, preventing the model from capturing complex patterns in the data, or because the test set happens to conform to the model’s processing pattern, resulting in good performance on the test set.

### 3.3. Model Interpretation and Feature Importance Analysis

**Figure 5:**
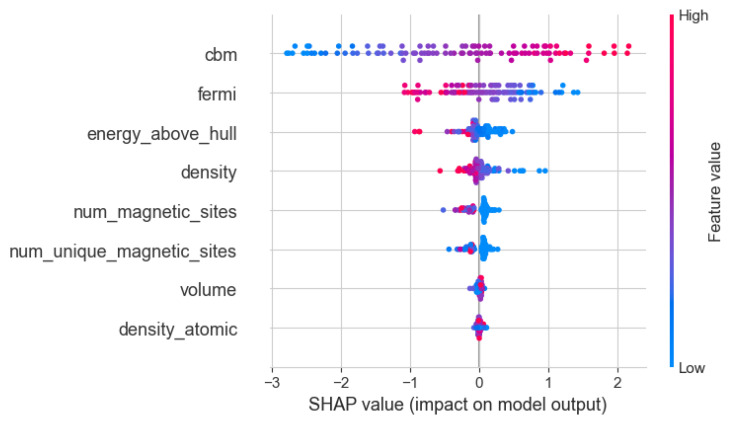
Overall feature importance plot for SHAP features.

**Figure 6:**
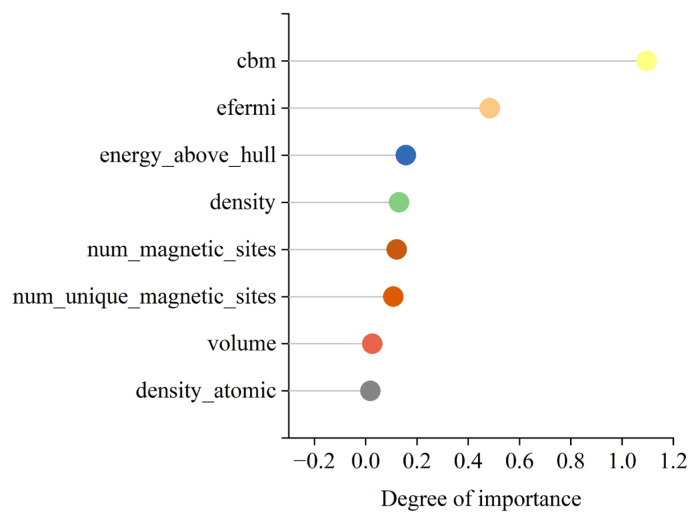
Importance ranking of features in SHAP analysis.

To enhance the transparency and interpretability of the model, the SHAP method, based on game theory principles, is adopted to illustrate the impact of input features on the AdaBoost model. Figure 5 and Figure 6 demonstrate the contribution of each input feature to the bandgap prediction capability during the training process of the AdaBoost model. The SHAP analysis results show that cbm exhibits significant importance in the prediction of the bandgap. Its SHAP value distribution is wide, with an absolute value as high as 1.09, indicating that cbm has a decisive influence on predicting the bandgap of silicon oxide materials. The SHAP value of efermi is 0.48, second only to cbm. Efermi reflects the filling state of electrons within the material and plays a crucial role in the material’s conductivity and stability. It is known that efermi has a strong inverse correlation with the bandgap. From the overall SHAP feature plot, it can be observed that the smaller the efermi value, the larger the bandgap of silicon oxide materials. The SHAP values of energy_above_hull, num_magnetic_sites, density, and num_unique_magnetic_sites range from 0.1 to 0.2, indicating considerable importance. These features affect the material’s thermodynamic stability, magnetic behavior, and physical properties, thus impacting the bandgap value. Other features have SHAP values less than 0.05, contributing less to the model’s predictions and primarily serving to fine-tune the model. Using SHAP analysis, it is known that the SHAP values of volume and density_atomic are less than 0.05, indicating a low contribution to the model. However, despite the low contributions of volume and density_atomic to the predictive model, they still exert some influence on the model. The atomic density and volume of materials are interrelated. When the volume of silicon oxide decreases (and atomic density increases), the distance between atoms decreases, leading to enhanced orbital overlap and stronger chemical bonds, which may result in a reduction in bandgap. Conversely, when the volume of silicon oxide increases, the spacing between atoms increases, reducing the strength of chemical bonds and narrowing the widths of the conduction and valence bands, thereby increasing the bandgap [47,48]. Using SHAP analysis, the interpretability and credibility of the model have been enhanced, providing a new perspective for intuitively understanding the relationship between material properties and the bandgap.

## 4. Conclusions

This paper aims to predict the bandgap of silicon oxide materials using machine learning techniques. Using systematic data analysis, feature engineering, and the construction of various machine learning models, this study investigates the applicability of five machine learning algorithms, including three boosting models (AdaBoost, GBR, and XGB) and two traditional models (random forest and SVR), in predicting the bandgap of silicon oxide materials. Additionally, the SHAP method is introduced to interpret and analyze the best predictive model, the AdaBoost model, to explore the impact patterns of different features on the bandgap. The following conclusions are drawn:

1. The R^2^ scores for AdaBoost, Random Forest, GBR, and XGBoost are all above 0.9, indicating a high degree of fit to the dataset and good predictive performance. The MAE and MSE are maintained around 0.2, demonstrating good stability and accuracy.
2. The enhanced machine learning model, AdaBoost, exhibits outstanding performance in prediction accuracy with a high R^2^ of 0.93, indicating excellent generalization ability for unseen data. Its MAE and MSE are as low as 0.21 and 0.17, respectively, placing it at the optimal level among the five predictive models and enabling highly accurate prediction of the silicon oxide bandgap.
3. The SHAP method, based on game theory, is used to analyze the AdaBoost model. The SHAP value distribution of cbm is wide, with an absolute value of 1.09, indicating that cbm has a decisive influence on predicting the bandgap of silicon oxide materials. The bandgap increases with the increase in cbm. The SHAP value of efermi is 0.48, second only to cbm, and also has a high contribution. Since efermi is negatively correlated with the bandgap, an increase in the efermi value leads to a decrease in the bandgap.

The study demonstrates that the AdaBoost-enhanced machine learning model can accurately predict the bandgap of lithium-ion battery materials made of silicon oxides. The study elucidates the relationship between material characteristics and bandgaps, providing theoretical guidance for the directed design of materials with specific bandgap properties. This has significant implications for the rapid screening and high-performance prediction of novel functional materials.

## Figures and Tables

**Table 1:** Selected data extracted from the dataset.

Number	Formula_Pretty	Band_Gap	Volume	Cbm	Density	Efermi	…	Num_Unique_Magnetic_Sites
1	Nb_2_Zn_2_SiS_5_O_24_	1.14	1526.501	1.62	2.901	0.79	…	0
2	Li_3_CrSiBO_7_	0.68	266.591	1.98	2.787	1.19	…	2
…	…	…	…	…	…	…	…	…
7636	Mg_48_Si_34_H_62_O_147_	4.29	3024.055	5.64	2.491	1.41	…	0
7637	Si_48_O_107_	0.03	3697.184	−2.43	1.374	−2.44	…	35

**Table 2:** Characterization of the data and corresponding presentation.

Number	Feature	Description
1	band_gap	The bandgap of a material, which is the energy difference between the top of the valence band and the bottom of the conduction band, is used to determine whether the material is an insulator, semiconductor, or metal.
2	volume	Volume of the material.
3	cbm	The energy of the conduction band minimum, corresponding to the lowest energy level of the conduction band in the band structure.
4	density	The density of the material, typically measured in grams per cubic centimeter (g/cm^3^).
5	density_atomic	Atomic density, calculated based on atomic mass and volume.
6	efermi	Fermi energy level, which is the energy level at which the probability of an electron being occupied is 50%.
7	energy_above_hull	The energy difference of the material relative to its most stable phase mixture; a negative value indicates thermodynamic stability, while a positive value indicates instability.
8	num_magnetic_sites	The number of atoms (or ions) in the material with non-zero magnetic moments.
9	num_unique_magnetic_sites	The number of different types of magnetic atoms (or ions) in the material.

**Table 3:** Test results for five regression models using 11 features as training samples.

Models	Band_Gap		
	R^2^	MAE	MSE
Random Forest	0.92	0.20	0.19
SVR	0.89	0.34	0.26
AdaBoost	0.93	0.21	0.17
GBR	0.91	0.24	0.21
XGB	0.91	0.23	0.20

Models

Band_Gap

R^2^

MAE

MSE

## Data Availability

The data used for the study were obtained from https://legacy.materialsproject.org/ (accessed on 13 June 2024).
